# An Emerging Role for Sigma Receptor 1 in Personalized Treatment of Breast Cancer

**DOI:** 10.3390/cancers15133464

**Published:** 2023-07-02

**Authors:** Taylor S. Robinson, Mahasin A. Osman

**Affiliations:** Department of Medicine, Division of Oncology, College of Medicine and Life Sciences, University of Toledo, Toledo, OH 43614, USA; taylor.robinson@rockets.utoledo.edu

**Keywords:** breast cancer biomarker, sigma receptor1, breast cancer, oligomerization, IQGAP1, Cdc42, Rac1

## Abstract

**Simple Summary:**

Breast cancer continues to be the number one cause of cancer mortality among women. Because breast cancer is a heterogenous disease there is a need for a more personalized approach to treatment. Such an approach requires the understanding of the molecular root cause of each cancer and the identification of the responsible molecule(s) or pathway(s). SigmaR1 is a receptor implicated in certain types of breast cancer and represents a promising target for a new generation of personalized treatments. However, there is a need for understanding its precise cellular role in order to target it effectively. This article reviews the current knowledge about SigmaR1 in breast cancer biology and potential treatment and proposes a new model as to how SigmaR1 operates within the cell in order to devise new effective ways for utilizing it in the clinic.

**Abstract:**

Despite the major progress in treating breast cancer, recurrence remains a problem and types such as triple-negative breast cancer still lack targeted medicine. The orphan Sigma receptor1 (SigmaR1) has emerged as a target in breast cancer, but its mechanism of action is unclear and hinders clinical utility. SigmaR1 is widely expressed in organ tissues and localized to various sub-cellular compartments, particularly the endoplasmic reticulum (ER), the mitochondrial-associated membranes (MAMs) and the nuclear envelope. As such, it involves diverse cellular functions, including protein quality control/ER stress, calcium signaling, cholesterol homeostasis, mitochondrial integrity and energy metabolism. Consequently, SigmaR1 has been implicated in a number of cancers and degenerative diseases and thus has been intensively pursued as a therapeutic target. Because SigmaR1 binds a number of structurally unrelated ligands, it presents an excellent context-dependent therapeutic target. Here, we review its role in breast cancer and the current therapies that have been considered based on its known functions. As SigmaR1 is not classified as an oncoprotein, we propose a model in which it serves as an oligomerization adaptor in key cellular pathways, which may help illuminate its association with variable diseases and pave the way for clinical utility in personalized medicine.

## 1. Introduction

Breast cancer is a heterogeneous collection of diseases that have been classified into at least four molecular subtypes that require a complex regimen of treatments [1]. Though much progress has been made in curing early-stage breast cancer, the development of resistance, recurrence, and metastatic disease remains the driver of the current rise in disease incidence and mortality [2]. Additionally, the triple-negative breast cancer (TNBC) subtype, itself is heterogenous and lacks targeted therapy [3]. Accordingly, approaches for personalized medicine have been highly sought after [2], including the identification of new molecular targets. Mounting evidence has presented Sigma receptor 1 (SigmaR1) as a potential target in the diagnosis and treatment of breast cancer.

SigmaR1 is an orphan, non-G protein, non-ionotropic transmembrane protein that regulates calcium signaling [4,5,6]. It is one of two types of sigma receptors present in the endoplasmic reticulum (ER) membrane [7]. SigmaR1 is widely known as an intracellular chaperone protein involved in numerous cellular functions including protein synthesis, protein folding, protein trafficking, unfolded protein response, and protein degradation [7]. Initially, SigmaR1 was believed to be a part of the opioid receptor family [8]. Later, radioligand binding experiments revealed the existence of two different types of sigma receptors with distinct subcellular distribution and ligand selectivity [9,10]. They have been named sigma-1 and sigma-2 receptors, although far more is known about SigmaR1 than SigmaR2. SigmaR1 was first cloned in 1996, and SigmaR2 was not cloned until 2017 from calf liver [7]. SigmR1 has been associated with variable human maladies, including addiction, analgesia, amnesia, depression, Alzheimer’s disease, stroke, HIV, and cancer [11,12,13,14,15,16,17,18,19]. It is not surprising that over the past several years SigmaR1 has been studied as a possible target for treatment for many human illnesses, particularly Alzheimer’s and cancer. However, despite intensive research, there is still little known about the mechanism(s) of SigmaR1 in these diseases to allow for targeted therapy in the clinic. This review will focus on the role of SigmaR1 in breast cancer and possible directions for investigating an underlying unifying function for SigmaR1 as an adaptor protein that may provide new insights into its multiple cellular roles and potential clinical use against breast cancer and other diseases.

## 2. Structure, Localization, and Function of SigmaR1

**Figure 1:**
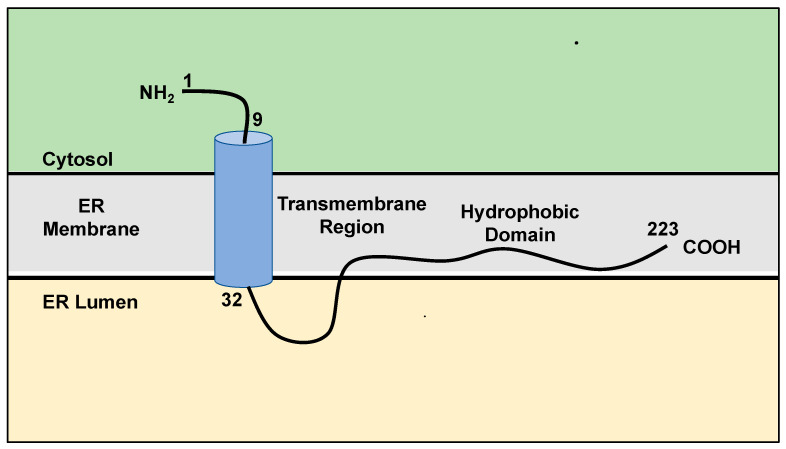
Schematic representation of the current knowledge of SigmaR1 structure. SigmaR1 is a transmembrane orphan receptor, ubiquitously localized at the endoplasmic reticulum (ER) and interfacing the mitochondrial membrane. It consists of a single transmembrane and a hydrophobic domain. See text for more details. Adapted from references [21,22].

SigmaR1 is found in the ER membrane and the mitochondria-associated membranes (MAMs) that interface the ER [7]. It was previously believed that SigmaR1 contains two transmembrane domains and an extramembrane loop of 50 amino acids long [20]. Recent structural studies, using protease protection, suggested a single 22 residues long transmembrane domain that is located near the nine-residue long N-terminal domain and is followed by a hydrophobic C-terminal domain at residues 32-223 associated with the ER membrane (Figure 1) [21,22].

Given this domain structure, detailed pharmacogenetic studies using disease-associated and created mutants of SigmaR1 will be an important approach to understanding its diverse and specific functions as well as identifying the locations of ligand-binding towards the effective clinical utility in cancer treatment. Such a genetic approach likely will also illuminate the determinants of sigmaR1 versatile locations.

Within the body, SigmaR1 is found in the central nervous system, as well as in peripheries such as the lung, kidney, liver tissues, and the immune, and endocrine systems [23]. Within the central nervous system, it is commonly found within the hippocampus and dorsal horn of the spinal cord but less so in the cerebellum [24]. Although SigmaR1 typically resides in the ER membrane and at MAMs, it has been found to translocate to other subcellular regions, such as the plasma membrane after stimulation by agonists [7], perhaps to regulate a variety of cellular functions such as ion channels [5]. Additionally, it was found in the nuclear envelope and the nucleoplasmic reticulum, a nuclear pocket made from the invagination of the nuclear envelope [24]. These versatile localizations suggest distinct functions and interactions with resident proteins that have yet to be fully defined.

Functionally, SigmaR1 has been associated with Ca^2+^ signaling and the ER stress response. It plays a major role in the unfolded protein response (UPR) [25] and binds cholesterol and restores lipid rafts [26]. SigmaR1 agonists inhibit Ca^2+^, K^+^, Na^+^, and Cl^−^ voltage-gated ion channels, which can be reversed by SigmaR1 antagonists [7]. When regulating Ca^2+^ at the MAMs, it forms a complex with immunoglobulin heavy-chain binding protein (BiP), which is a major ER chaperone protein [27]. When calcium levels are low or SigmaR1 is stimulated via ligand binding, SigmaR1 dissociates from the complex causing increased calcium signaling by inositol triphosphate receptor (IP3Rs), an ER-resident membrane protein and thus acts as Ca^2+^-sensitive chaperone [4]. Decreasing SigmaR1 levels was suggested to promote ER-stress response, leading to apoptosis [27]. Dreser et al. evaluated the known SigmaR1 E102Q mutation associated with Amyotrophic Lateral Sclerosis (ALS) and SigmaR1-mediated neurodegeneration to study the effects of SigmaR1 on protein homeostasis, using immunohistochemistry, immunocytochemistry, and mitochondrial toxicity assays [28]. The study revealed that E102Q-SigmaR1 protein aggregates concentrated in the ER and nearby compartments in transfected cells, causing structural changes in the mitochondria, the nuclear envelope, and ER, as well as altering calcium homeostasis and inducing defective autophagy. Accordingly, SigmaR1 has been investigated in this context, as the role of calcium homeostasis and ER stress in breast cancer has been shown and sought after as a potential clinical target in breast cancer treatment [29,30], which is discussed below in more detail.

## 3. The Role of SigmaR1 in Breast Cancer

SigmaR1 has been found to be upregulated in a number of cancers including lung cancer, breast cancer, glioblastoma, esophageal cancer, pancreatic cancer, prostate cancer, and liver cancer [31,32,33,34,35,36,37,38]. Several studies showed that SigmaR1 is highly expressed both at the gene and protein levels in breast cancer patient tissues and is associated with a worse prognosis and poor survival outcomes [33,39,40]. Whether this association represents a correlation, or a causality is still awaiting mechanistic studies. Of these studies, Borde et al. indicated that not only was SigmaR1 upregulated in breast cancer cell lines, but it was especially upregulated in triple-negative breast cancer cell lines [40]. An earlier study of 95 patients with operable primary breast cancers by Simony-Lafontaine et al. [39] reported a link between SigmaR1 and human sterol isomerase (hSI). This study revealed a correlation between the expression levels of SigmaR1, hSI and the antiapoptotic B-cell leukemia/lymphoma 2 (Bcl2) protein, and that patients with no SigmaR1 expression in the presence of hSI had a poorer disease-free survival [39]. In a study conducted on 109 normal, benign, and cancerous breast tissue specimens to determine SigmaR1 expression level in breast cancer by using immunohistochemistry and qRT-PCR, SigmaR1 mRNA overexpression was present in 60% of invasive cancers, 41.5% of in situ cancers, 75% of ductal hyperplasias, and 33% of normal breast tissue [33]. The same study found that the concentration of SigmaR1 in the tissue samples did not correlate with patient survival expectations [33]. Thus, it appears that the connection between SigmaR1 expression level and disease status is context-dependent and further mechanistic studies of its role are needed, particularly that the mRNA levels may or may not correlate with or reflect the protein levels. Additionally, nothing thus far has been shown about the likely effects of protein post-translational modification on SigmaR1’s role in breast cancer.

Mechanistic studies into how SigmaR1 promotes cancer cell proliferation identified several signaling routes, at the heart of which are Ca^2+^ signaling and ER stress. A study by Gueguinou et al. reported that SigmaR1 was necessary to increase calcium influx by provoking the calcium-activated potassium channel, SK3 and Orai1, a voltage-independent calcium channel in breast cancer cells [41]. Biochemical and confocal microscopic studies showed that SigmaR1 physically binds to SK3 channels and that inhibiting SigmaR1 reduced SK3 and Orai1 expression in breast cancer cells. Increased levels of SigmaR1 were found in breast cancer tissue samples and were correlated with tumor grade. Analyzing nearly 5000 patients with breast cancer revealed that the increased expression of both SigmaR1 and Orai1 channels was linked to poorer overall survival rates [41]. Computational modeling following induction of ER stress by antiestrogens in breast cancer cells identified several genes associated with the UPR transcription factor X-box binding protein 1 (XBP1) including BCL2, the proapoptotic BCL2-interacting Killer (BIK) and the Nuclear Factor Kappa B (NF-κB) [29]. These were believed to integrate with autophagy and apoptosis to determine breast cancer cell survival decisions [30].

Taken together these reports paint a complex picture of the cellular role of SigmaR1, and suggest that its effect on breast cancer is context-dependent, and likely acting through yet-to-be-identified oncoprotein(s) and/or other mechanisms that favor cancer cell proliferation or survival such as modulation of energetic, apoptotic and autophagic pathways.

## 4. Towards Targeting SigmaR1 in Breast Cancer

Despite the unclear function and the complex cellular role, active current research has been directed to investigating the potential of targeting SigmaR1 in breast cancer, particularly the TNBC subtype that lacks biomarkers. Pharmacologic studies appear to support the notion that targeting SigmaR1 could be a viable therapeutic approach. While SigmaR1 is not considered an oncoprotein, cancer cell lines require a functional SigmaR1 for proliferation and SigmaR1 inhibition has been found to prevent proliferation leading to apoptosis [42,43]. Thus, intensive research has been conducted to leverage the vast small molecule inhibitors of SigmaR1 in the quest for effective anticancer treatments.

Interestingly, SigmaR1 binds an array of structurally unique compounds (Table 1). Despite being orphan, the receptor appears to be a ligand-regulated protein where ligand binding elicits cellular responses often via the regulation of voltage-gated K+ channels, intracellular calcium levels, and neurotransmitter release, including acetylcholine and glutamate [44].

While this array of diverse ligands provides tools for mechanistic studies and likely therapeutics in personalized medicine, some of the most common SigmaR1 antagonists relevant to this discussion are the hormone progesterone and haloperidol (Haldol) [24,52]. Haldol is a first-generation FDA-approved antipsychotic widely used for treating schizophrenia [66]. Although Haldol binds both to SigmaR1 and dopamine receptors with similar affinities, the Haldol metabolite preferentially binds irreversibly to SigmaR1 rather than dopamine receptors [67]. Due to its relationship with SigmaR1 and the role it plays in various cancers, including breast cancer, intensive research has been conducted into the use of Haldol and its analogs as anticancer therapeutics. Haldol was reported to inhibit MCF7 cells over-expressing the ABCG2 transporter breast cancer resistance protein (BCRP) at >100 μM [68], while Haldol analogs such as SYA013 and SYA014 were reported to “halt” cell proliferation in the TNBC MDA-MB-231 and MDA-MB468 cell lines at 2–10 μM [69,70]. The molecular target and mechanisms of action of Haldol or its analogs in cancer cell inhibition remain unknown. The identification of such target(s) is crucial to devising more personalized analogs and dosing regimens while avoiding potential adverse side effects of Haldol such as dyskinesia or those that may adversely affect women with established breast cancer as suggested in the study by Rahman et al., [71]. As discussed above, SigmaR1 is either over- or under-expressed or found associating with distinct molecules in cancer, indicating the need for a more personalized approach to utilizing it clinically. In support of this notion, the evaluation of six different cell lines by Western blot indicated the presence of SigmaR1 proteins in four cell lines, including MDA-MB-435, BT20, MDA-MB-361, and MCF7. The treatment of these cell lines with high concentrations of Haldol showed growth inhibition in these cell lines [33], thus presenting SigmaR1 potential and necessity in personalized medicine. A recent study suggested that Haldol targets the scaffold oncoprotein IQ motif-containing Ras GAP-like protein1 (IQGAP1), altering its signaling by modulating protein-protein interactions, and inhibits cell proliferation in several TNBC cell lines [72]. Accordingly, it is likely that SigmaR1 engages oncoproteins such as IQGAP1 to initiate and sustain the oncogenic events. More research is underway to determine whether the IQGAP1-SigmaR1 axis is a direct target of Haldol in breast cancer as well as brain cancer.

Other SigmaR1 antagonists have also been studied for cancer cell inhibition. A recent study by Borde et al., reported that treatment in different breast cancer cell lines, including a high SigmaR1 expressing TNBC and an estrogen receptor (ER) + (low-SigmaR1 expressing) with the SigmaR1 antagonist 1-(4-iodophenyl)-3-(2-adamantyl)guanidine (IPAG) activated the UPR in both cell lines and caused aggregation and colocalization of the receptor with the ER-stress marker binding immunoglobulin protein (BiP) in the tamoxifen-resistant LY2 cells [40]. Oflaz et al. used single-cell fluorescence microscopy to evaluate real-time ion and metabolic fluxes in A549 lung adenocarcinoma and MC57 breast cancer cell lines [73]. When treated with the prototype SigmaR1 agonist (+)-SKF10047, it was noted that the bioenergetics of the mitochondria increased, while simultaneously decreasing aerobic glycolysis of the cells. In contrast, the selective SigmaR1 antagonist BD1047 did not elicit a change to mitochondrial bioenergetics but did have a notable increase in reliance on aerobic glycolysis [73].

Similarly, SigmaR1 has been a target of the search for treatment in other types of cancer that can be translated to treating breast cancer. Thomas et al., using in vitro and in vivo studies, reported that SigmaR1 inhibitors significantly inhibited prostate tumor growth related to loss of androgen receptor (AR) or the expression of its truncated splice form ARV7 [74]. This finding may be useful in treating variants of TNBC defined by the expression of the ARV7, which lend resistance to anti-AR treatments. Recent studies have identified the presence of ARV7 in a large number of breast cancer tissues [75,76]. Using RNAseq and DNAseq analyses in a large breast cancer cohort, Ferguson et al. [76] reported the detection of ARV7 in primary, metastatic and recurrent breast cancer tissues and proposed testing for ARV7 as a predictive biomarker for AR antagonists. This testing may be useful for assessing treatment options that include SigmaR1 inhibitors in the future.

SigmaR1 regulates reactive oxygen species (ROS) via the ferroptosis component nuclear-erythroid 2-related factor 2 (NRF2) and was found to be both up-regulated and translocated away from the nucleus in hepatocellular carcinoma cell lines in response to the ferroptosis inducer sorafenib [27]. The inhibition of SigmaR1 in these cell lines resulted in increased cellular death by sorafenib-induced ferroptosis both in vitro and in vivo, suggesting that SigmaR1 shields hepatocellular carcinoma cells against sorafenib-induced apoptosis [27]. This strategy is likely useful in a subset of breast cancers.

Overall, these studies illustrate the diversity of SigmaR1 functional interactions and differential influence on the different cancer cell types. Furthermore, the versatility of the agonists and antagonists that influence SigmaR1 activity (Table 1) suggests a complex role for this receptor and requires further mechanistic analyses of its function in order to facilitate clinical utility in the personalized management of heterogenous diseases such as breast cancer. Below we consider a model gleaned from bacteria and biophysical studies conducted on SigmaR1 to illuminate the role of this simple yet complex receptor, and potentially provide a unifying action mechanism for its function and dysfunction in disease states.

## 5. Perspective: SigmaR1 as an Adaptor Protein in Key Cellular Functions

Thus far, despite the accumulated wealth of information about SigmaR1 roles and interactions, its exact cellular mechanism of action remains elusive, which hinders the development of effective targeted therapies. Here, we propose a model in which SigmaR1 serves as an adaptor protein that mediates the oligomerization of multiprotein machines in diverse cellular functions such as protein synthesis, folding, transport, and signal transduction pathways in which it was implicated. Naturally, the dysfunction of such machines would lead to the myriad of complex diseases associated with SigmaR1 dysregulation or modulation by diverse ligands.

**Figure 2:**
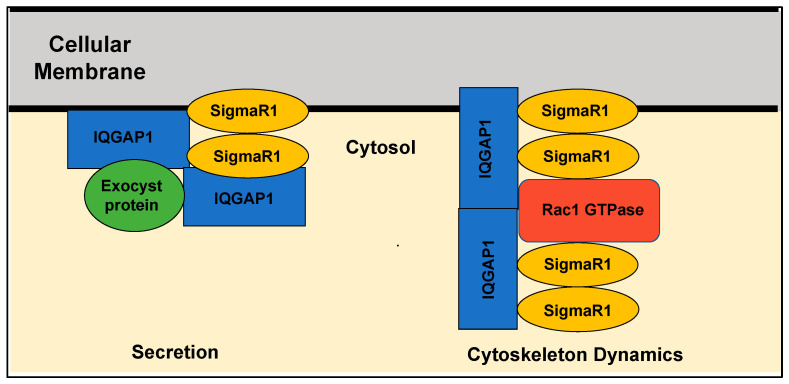
A proposed model for Sigma1 as an oligomerization adaptor in signaling pathways controlling key cellular functions. Gleaning from biophysical data [77,78] and bacterial models [82], it is proposed here that SigmaR1 serves as an oligomerization adaptor that forms higher-order complexes in key pathways and involves GTPases such as Rac1 and scaffold molecules such as IQGAP1 to ensure specificity of complex formation and thus cellular functions of common molecules. The cellular membrane represents plasma or any organelle membrane where SigmaR1 is located. Secretion depicts vesicular-mediated exocytic activities, including neurotransmitters or Golgi transport.

Models of SigmaR1 actions predict that ligand binding to the receptor modulates its oligomerization status, whereby some ligands stabilize the monomeric form and others facilitate the formation of dimeric or tetrameric complexes [77,78]. A recent biophysical study suggested that the E102Q mutation associated with familial amyotrophic lateral sclerosis (ALS) disrupts the higher-order oligomerization of SigmaR1, thus leading to its dysfunction [79]. Cells use oligomerization for allosteric regulation of proteins to control activities and specify diverse functions carried out by common proteins [80]. Studies from several bacterial species provide clear evidence that adaptor proteins participate in organizing higher-order oligomerization of interacting proteins, not only to control their activities but also their specificity towards binding partners [81]. For example, the HSP100/ClpC of *Bacillus subtilis* controls key cellular steps, including stress response [82] and protein quality control [83,84]. The activity of the ClpC requires the presence of the adaptor protein MecA, whereby an active substrate-recognizing higher oligomer consisting of ClpC and MecA is formed in the presence of ATP [85]. Given that SigmaR1 is a unique orphan transmembrane receptor that undergoes oligomerization, regulates the activity of numerous cellular proteins, and is modulated by a plethora of unrelated small molecules as discussed above, we propose that it operates analogously to allosteric regulatory adaptor proteins such as the MecA system whose function is regulated, at least in part, by oligomerization (Figure 2). Investigating this adaptor model within the context of SigmaR1’s diverse functions would provide a framework for devising effective personalized therapies for individual conditions and for understanding the biology of SigmaR1.

Another missing piece of information hindering the development of targeted therapies against SigmaR1 is the specific signal transduction pathway(s) controlling the protein synthesis and secretion role of SigmaR1 and how they are affected by the various ligands [86]. Likely regulatory candidates are the members of the Ras superfamily of small G-protein enzymes (GTPases). Indeed, the interaction of SigmaR1 and Rac1 GTPase in bovine membrane mitochondria was suggested to induce oxidative stress and prevent apoptosis and autophagy [87]. Rac1 is widely known for its regulation of the actin cytoskeleton and vesicular traffic and has been implicated in breast cancer [88]. The oncoprotein IQGAP1 is a scaffold signal regulator acting both upstream and downstream of several members of the Ras superfamily GTPases, including Rac1, which has an essential underlying role in secretion and transport, and it binds different types of receptors [89,90,91]. IQGAP1 undergoes oligomerization into dimers that bind actin filaments and organizes them into thin bundles [92]. Biochemical studies demonstrated IQGAP1 oligomerization via N-terminus amino acids 216-683 or in interactions with Cdc42 or Rac1 GTPases [93]. Clearly, it appears that oligomerization regulates IQGAP1 activity in many of its diverse functions, but how this oligomerization is mediated in a context-dependent manner remains unclear. Recently, Haldol, a SigmaR1 antagonist (Table 1), was found to modulate IQGAP1 signaling in TNBC cell lines, and an association with SigmaR1 was detected both in vitro in many cell lines and in vivo [52]; manuscript in preparation). Furthermore, separate studies have demonstrated a striking commonality between IQGAP1 and SigmaR1 functions. Like SigmaR1, IQGAP1 promotes ROS via Rac1 GTPase [94], and studies in mouse models have implicated IQGAP1 in memory and cognitive deficits [95,96]. Biochemical and patch-clamp studies showed that IQGAP1 [96] and SigmaR1 [97] interact with the ionotropic glutamate N-methyl-D-aspartate receptor (NMDR) in the neurons and influence synaptic processes. Additionally, IQGAP1 localizes to the same subcellular regions as SigmaR1, including the plasma membrane, the nuclear envelope and the ER, and is involved in similar disease outcomes such as cancer and diabetes [98]. Taken together, these findings may help explain why SigmaR1 promotes oncogenic growth while itself is not an oncoprotein and provide avenues for utilizing SigmaR1 in personalized medicine. Thus, much research lies ahead to mechanistically utilize the SigmaR1-IQGAP1 axis as a precision target in treating breast cancer. Thus much exciting research remains ahead to decipher the exact role of SigmaR1 in cancer and developing potential treatments.

## 6. Conclusions

In summary, SigmaR1 is a small receptor with variable cellular functions, and is associated with a wide range of human diseases, including breast cancer. While its cellular and disease mechanisms are still emerging, this role can be explained by the variety of the protein-protein interactions that SigmaR1 forms combined by a likely function as an oligomerization adaptor molecule that mediates the assembly of permutation of multiprotein machines. Key cellular processes are generated by multiprotein complexes, often made up from the same proteins in different combinations, disfunction of such machines leads to the myriad of diseases such as those attributed to SigmaR1. Much research is required to test and verify this hypothesis and to define the mechanism of action of SigmaR1 in health and disease.

## Figures and Tables

**Table 1:** A Selection of Common SigmaR1 Ligands.

Compound Name	Function	Clinical Use	References
**Chloroquine**		Anti-malarial drug	[45]
**BD1047**	Antagonist	Has antipsychotic properties, potential use as a neuropathic pain treatment (in phase II clinical trials)	[46,47]
**BD1063**	Antagonist	Reduces effects of cocaine, MDMA	[46,47]
**Cannabidiol (CBD)**	Antagonist	Treatment for pain, cancer symptoms and holds potential for Alzheimer’s, Parkinson’s, stroke, epilepsy, neuropsychiatric disorders	[48]
**E52862**	Antagonist	Potential treatment for neuropathic pain	[49]
**Dehydroepiandrosterone**	Agonist	Anti-aging therapy, used to treat depression and menopause symptoms	[50]
**Haloperidol**	Antagonist	Antipsychotic	[24]
**NE-100**	Antagonist	Commonly used to study SigmaR1	[51]
**Progesterone**	Antagonist	Important hormone of the menstrual cycle and helping maintain the early stages of pregnancy	[45,52]
**Sertraline**	Antagonist	Selective serotonin reuptake inhibitor used to treat depression, OCD, PTSD, and panic attacks	[53]
**Verapamil**	Antagonist	treats cardiac issues including angina, heart rhythm problems, and hypertension	[54]
**Cocaine**	Agonist	Central nervous system (CNS) stimulant	[11,12,34,55]
**Choline**	Agonist	Triggers IP_3_-evoked Ca^2+^ release	[56]
**Donepezil**	Agonist	Dementia medication	[57,58]
**Dextromethorphan**	Agonist	Cough suppressant	[59]
**N, N-dimethyltryptamine (DMT)**	Agonist	hallucinogen	[60]
**Fluvoxamine**	Agonist	Selective serotonin reuptake inhibitor used to treat depression and OCD	[61]
**Ifenprodil**	Agonist	Cerebral vasodilator	[62]
**Memantine**	Agonist	Used to treat Alzheimer’s disease symptoms	[47]
**Methamphetamine**	Agonist	Central nervous system (CNS) stimulant	[45]
**Pentazocine**	Agonist	Opioid pain medication	[63]
**SA4503 (Cutamesine)**	Agonist	Anti-amnesia properties, inhibits angiotensin II-induces cardiomyocyte hypertrophy	[64]
**SKF10,47**	Agonist	Opioid analgesic	[65]
**4-(N-benzylpiperidin-4-yl)-4-iodobenzamide (4-IBP)**	Agonist	Decreases migration of human cancer cells in vitro	[36]
